# Socioeconomic Factors Associated With Nonunion in Scaphoid Injuries: A Novel AI Model

**DOI:** 10.1016/j.jhsg.2026.101176

**Published:** 2026-11-04

**Authors:** Muhammad H. Hamdan, Aidan M. Lynch, Jordan Bauer, Rohan V. Rajan, Rajendra Singh, Joel V. Ferreira, Anthony Parrino, Craig M. Rodner

**Affiliations:** ∗ Department of Orthopedic Surgery, University of Connecticut, Farmington, CT

**Keywords:** Area deprivation index, Neural network, Nonunion, Scaphoid fractures, Social determinants of health

## Abstract

**Purpose:**

Scaphoid fractures, the most common carpal fractures, are prone to nonunion because of their retrograde blood supply. Delayed presentation increases the risk of nonunion and progression to scaphoid nonunion advanced collapse. Social determinants of health, as measured by Area Deprivation Index (ADI), Social Vulnerability Index (SVI), and Child Opportunity Index (COI), can influence timely presentation, increasing the risk of nonunion. This study aims to develop a novel artificial intelligence model to predict scaphoid nonunion rates using socioeconomic indices alone.

**Methods:**

A retrospective review identified 107 patients with suspected scaphoid fractures (January 2018–June 2025) who presented within 5 years of injury. National ADI, SVI, and COI scores were obtained. A binomial regression was used to validate ADI, SVI, and COI scores to predict nonunion status. A multilayer neural network was trained on the indices to predict nonunion status. Model performance was evaluated with area under the curve, and Youden’s J statistic was maximized to determine sensitivity and specificity.

**Results:**

Higher ADI (OR = 1.029; 95% CI, 1.009–1.049) and SVI (OR = 1.020; 95% CI, 1.006–1.035), and lower COI (OR = 0.986; 95% CI, 0.974–0.998) were independent predictors of nonunion on presentation, validating their usage. The neural network had an area under the curve of 0.879, and at a threshold of 0.21 (J = 0.707), the model achieved a sensitivity of 95.7%, specificity of 75.0%, precision of 0.512, and an F1 score of 0.667.

**Conclusions:**

Our artificial intelligence model predicts nonunion status based purely on social determinants of health, emphasizing their influence on scaphoid fractures. Addressing these disparities is essential to improve timely access to care for injuries such as scaphoid fractures and may be crucial to other timely orthopedic fractures.

**Type of study/level of evidence:**

Prevalence IIIb.

Scaphoid fractures are the most common carpal fractures, predominantly impacting young, active males, often occurring as a result of a fall on an outstretched arm.^1^, ^2^, ^3^, ^4^ These fractures are characterized by their location: proximal (25% of cases), waist fractures (65% of cases), and distal pole fractures (10% of cases). The scaphoid’s unique retrograde blood supply and anatomical positioning make it, especially the proximal pole, particularly susceptible to nonunion and avascular necrosis if not treated in a timely manner.^2^, ^5^, ^6^, ^7^, ^8^ Nonunion of the scaphoid can lead to scaphoid nonunion advanced collapse (SNAC) and subsequent degenerative arthritis of the wrist, with proximal pole fractures particularly prone to nonunion and avascular necrosis.^7^ Moreover, smoking increases the risk of nonunion by approximately 20%, and dislocated fractures ≥1 mm also have a higher nonunion risk if treated nonsurgically.^6^, ^9^, ^10^ Therefore, individuals who experience delays in diagnosis and appropriate management of these fractures are at higher risk of nonunion and its subsequent complications.

Initial diagnosis of suspected scaphoid fractures, such as in patients with snuffbox pain after a fall on an outstretched wrist, is determined by clinical examination and radiographic imaging. Initial radiographic imaging in the presence of snuffbox tenderness should be treated with immobilization followed by repeat imaging a week or two later. If plain films remain inconclusive, but snuffbox tenderness persists, advanced imaging such as magnetic resonance imaging (MRI) or computed tomography (CT) scan can be used to assess for an occult scaphoid fracture.^4^, ^6^

Individuals without insurance or on public health insurance routinely experience delays in seeking orthopedic care and, thus, present with more advanced conditions.^11^, ^12^, ^13^, ^14^, ^15^ Although this association is strong, insurance status does not fully encapsulate a patient’s social determinants of health (SDoH).^16^ SDoH can be quantified into measurable and comparative tools, including the Area Deprivation Index (ADI), the Social Vulnerability Index (SVI), and the Child Opportunity Index (COI). ADI is an index that stratifies neighborhoods based on income, education, employment, and housing quality.^17^ Although Lynch et al^18^ have previously shown that state and national ADI are predictors of nonunion rates and delayed presentation for scaphoid fractures, there is a lack of studies comparing multiple SDoH indices, age, insurance status, and smoking status to create a robust predictor model, as our study aimed to do.^18^, ^19^ Moreover, this article presents a novel model for the risk factors for presenting with a scaphoid nonunion on initial presentation.

## Materials and Methods

This study is a retrospective chart review of patients who were seen at a single academic medical center for scaphoid fractures with an International Classification of Diseases, Ninth and Tenth Revisions (ICD-9 or ICD-10, respectively) code S62.0 encompassing scaphoid fracture diagnoses between January 1, 2018, and June 30, 2025. Patients were excluded if further imaging (such as an MRI or a CT scan) did not reveal a scaphoid fracture. Patients who presented more than 5 years after injury were excluded to minimize bias from extreme outliers. These cases, some presenting up to 50 years after injury, were considered atypical and not representative of contemporary diagnostic or treatment delays. Institutional Review Board approval was obtained, and data were coded for Health Insurance Portability and Accountability Act compliance.

Data collected included the following: nine-digit zip code, age at presentation, time from injury to presentation, and presence of radiographic nonunion. Imaging protocols were standardized at our institution, with all patients receiving wrist radiographs and advanced imaging (MRI or CT) when initial films were inconclusive. Using the nine-digit zip code, a state ADI score was assigned to each patient using the University of Wisconsin School of Medicine’s ADI tool.^17^ With respect to demographic information, ADI is a comprehensive tool that has been demonstrated to be more predictive of SDoH than individual-level measures such as race or ethnic background.^20^ ADI is scored from 1 to 100, with 1 being low deprivation and 100 being high deprivation. Using the nine-digit ZIP code, national SVI and COI were determined for each patient.^21^, ^22^ SVI considers socioeconomic status, household characteristics, racial/ethnic minority status, housing type, and transportation.^21^ SVI is a percentile ranking from 0 to 1, with 0 being low deprivation and 1 being high deprivation, but SVI values were multiplied by 100 to adjust the data range from 0 to 1 to 0 to 100 to match the scale of National ADI. COI factors in the quality of schools, housing, food access, parks, clean air, and economic opportunities.^23^ COI is scored 1-100, with a higher score indicating increased access and less deprivation. Each of these indices can be used on a national or state level when comparing to other neighborhoods, with national scores being preferred for their generalizability. The factors used for analysis included age, insurance status, COI, SVI, and ADI. Using IBM SPSS, a binomial regression was used to determine what factors best predict nonunion status to validate these indices for model creation. This was followed by training a novel multilayer perceptron neural network to predict nonunion using the previously mentioned factors. The architecture of the model was set automatically by the SPSS software, resulting in one hidden layer with four nodes. The model used one hidden layer with a hyperbolic tangent activation function and was trained using the scaled conjugate gradient algorithm. Data were split into training and testing sets using SPSS’s default settings of 70/30. Model performance was evaluated using area under the curve (AUC) and classification metrics. Predicted probabilities were converted to binary classifications using an empirically optimized threshold, selected by maximizing Youden’s J statistic, to determine sensitivity and specificity. Significance was defined as *P* < .05.

## Results

One hundred sixty-eight total patients were identified by ICD-9 and ICD-10 queries for scaphoid fracture (S62.0) who presented between January 1, 2018, and June 30, 2025. From this cohort, 52 were excluded for not having a scaphoid fracture after advanced imaging, and nine were excluded for presenting after 5 years, giving a final study population of 107 patients. The final study population had an average age of 29.0 ± 15.4 years and was 73.8% male.

Univariable binary logistic regressions were used to identify the factors that best predict nonunion status. Higher National ADI (OR = 1.029, *P* = .004) and adjusted National SVI scores (OR = 1.020, *P* = .005) were associated with increased odds of nonunion, whereas higher COI scores were associated with decreased odds of nonunion (odds ratio [OR] = 0.986, *P* = .020; Table 1). Age and insurance status were not significantly associated with nonunion.

**Table 1:** Binary Logistic Regression for Nonunion Status∗

Predictive Measure	Odds Ratio (95% CI)	*P* Value
**National ADI Score**	**1.029 (1.009–1.049)**	**.004**
**National SVI**†**Score**	**1.020 (1.006–1.035)**	**.005**
**Child Opportunity Index**	**0.986 (0.974–0.998)**	**.020**
Insurance Status	1.213 (0.873–1.686)	.250
Age	1.003 (0.981–1.027)	.770

∗ Statistically significant results are bolded.

† Adjusted SVI score.

A multilayer perceptron neural network was trained to predict nonunions using socioeconomic factors (Fig. 1). The training sample contained 89 patients (83.2% of the patient population), whereas the testing population contained 18 patients (16.8%). Overall, the model was 82% correct for the training set and 83.3% for the testing sample. The model achieved an area under the receiver operating characteristic curve (AUC) of 0.879 (Fig. 2). The threshold of 0.21 was selected based on Youden’s J statistic (J = 0.707) to balance sensitivity and specificity. At this threshold, the model achieved a sensitivity of 0.957, specificity of 0.750, precision of 0.512, and an F1 score of 0.667.

**Figure 1:**
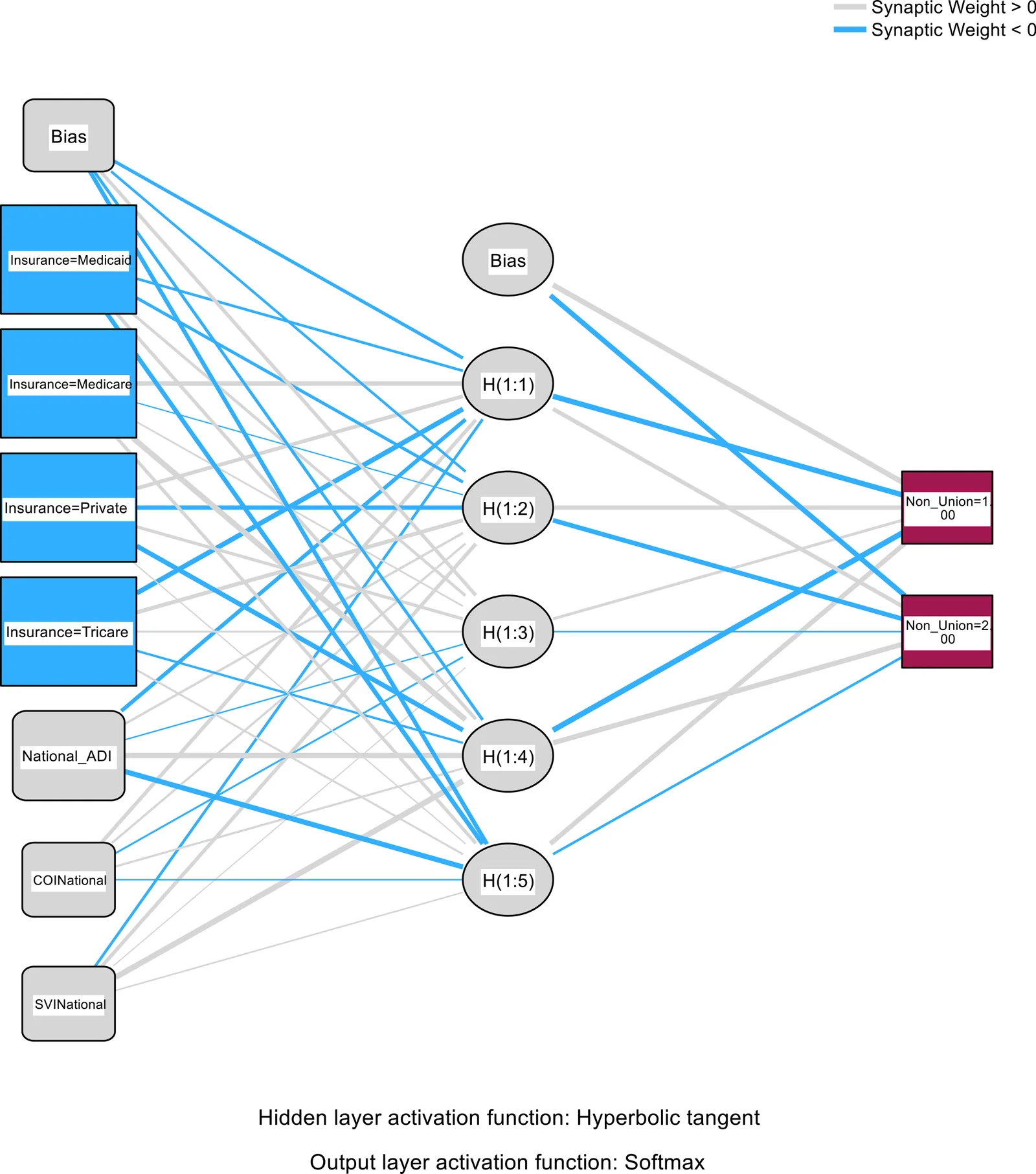
Architecture of the multilayer perceptron neural network trained and tested on the data set.

**Figure 2:**
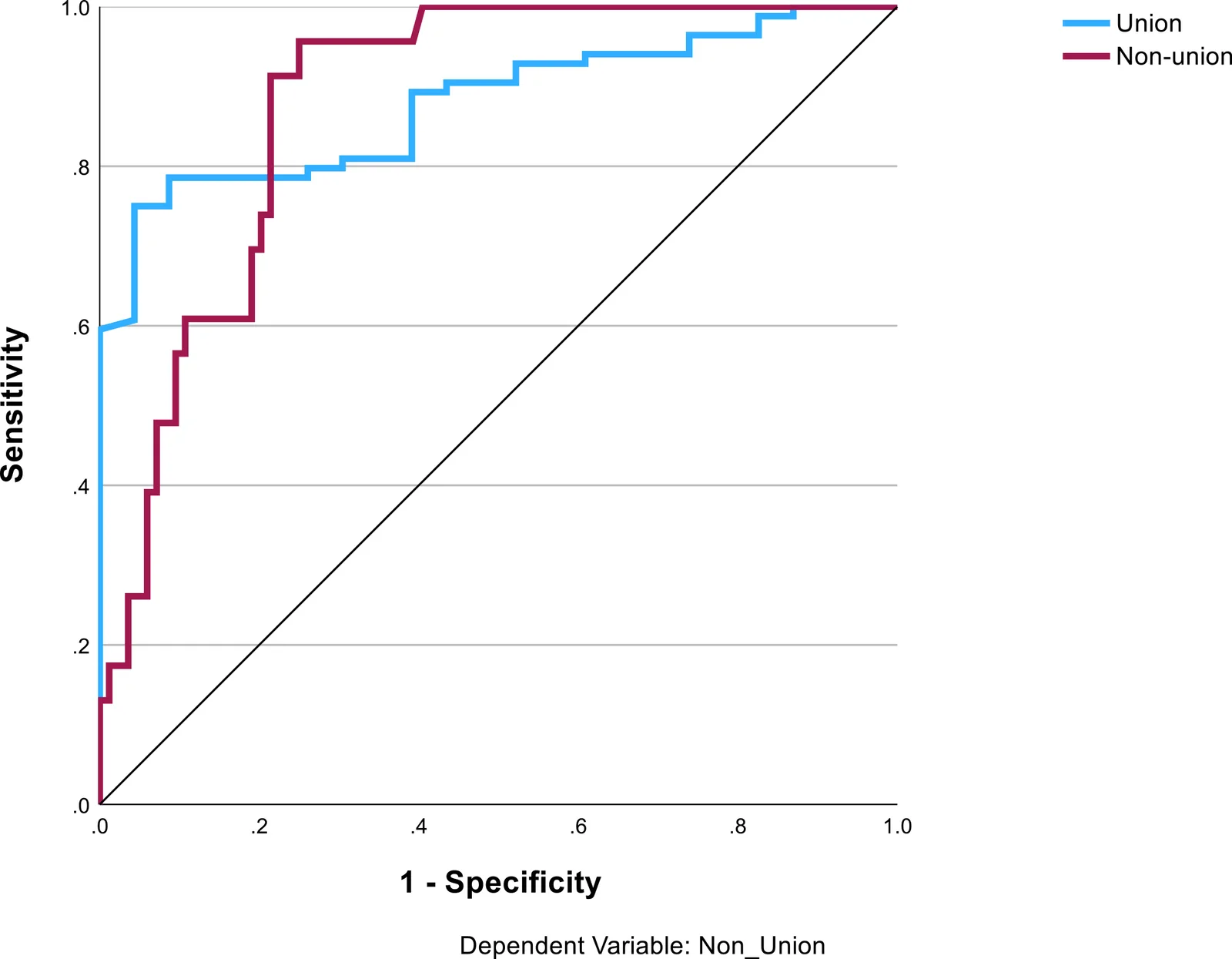
Receiver operating characteristic curve of the multilayer perceptron neural network for predicting nonunion and unions.

Variable importance analysis revealed that insurance status was the most influential predictor (normalized importance = 100.0%), followed by National ADI (73.8%), National COI (52.6%), and National SVI (52.6%; Table 2).

**Table 2:** Variable Importance and Normalized Importance in the Multilayer Perceptron Neural Network Model for Predicting Nonunion

Predictive Measure	Variable Importance	Normalized Importance
Insurance Status	0.359	100.0%
National ADI Score	0.265	73.8%
National SVI Score	0.188	52.6%
Child Opportunity Index	0.188	52.6%

## Discussion

This retrospective study leveraged national socioeconomic data to create a novel model aimed at predicting nonunion status. Across 107 patients, these data provide evidence that patients with scaphoid fractures in the most deprived communities (high ADI and SVI, low COI) had increased odds of nonunion compared to patients from less deprived communities. A key strength of this study is the integration of multiple SDoH factors into a single prediction model. Although prior work has examined individual socioeconomic measures, this multilayer perceptron neural network using multiple measures demonstrated good discriminative performance (AUC = 0.879) to estimate nonunion risk. Given that scaphoid nonunions are a relatively uncommon outcome, sensitivity and specificity are more informative than accuracy alone. Using a probability threshold of 0.21, the model achieved a sensitivity of 95.7% and specificity of 75.0%, suggesting that the model may be more useful as a screening tool to identify high-risk patients rather than as a definitive diagnostic tool. This is because imaging and clinical evaluation provide a definitive diagnosis, whereas social determinants of health amplify the risk of nonunion.

Nonunions generally lead to worse outcomes because of the development of SNAC wrist arthritis and subsequent degenerative arthritis of the wrist.^6^, ^24^, ^25^, ^26^ Acute, stable scaphoid fractures are usually managed with nonsurgical cast immobilization or percutaneous internal screw fixation, whereas nonunion fractures typically require an open fixation with vascularized or nonvascularized bone grafts.^6^ If surgical fixation fails to unionize or the treatment delay is more significant, patients are at risk of developing SNAC wrists. The longer SNAC wrists are left untreated, the more extensive salvage reconstructive procedures or arthrodesis may become necessary, significantly limiting wrist motion and grip strength.^6^ Per Lynch et al,^18^ a delay in treatment is directly related to a higher ADI tercile, which suggests patients in higher ADI terciles are more likely to need higher risk surgical interventions. Reducing the barriers to care that delay the time to treatment of patients from more deprived communities is essential to promoting earlier, less invasive intervention while preventing long-term complications.

The indices used provide complementary but unique insight into the biggest drivers of a patient's delayed presentation following a hand injury. ADI looks at income/employment, education, housing quality, and household characteristics. SVI also looks at socioeconomic status, but expands to household composition and disability, racial/ethnic minority status, English proficiency, and housing type. Lastly, COI explores education, health, and environmental safety (including food and air quality), and socioeconomic status. We also examined insurance status, as even with access to transportation or green spaces, insurance status, specifically being on Medicaid, can severely limit access to care.^27^ Overall, the results provide insight that income, education, employment, and transportation all play a strong role in how fast a patient can present to the orthopedic office.

Several prior studies have demonstrated that social determinants of health contribute to higher rates of orthopedic injuries, particularly high-energy traumas, along with delays in accessing care, worse function at presentation, and higher complication rates.^28^, ^29^, ^30^, ^31^, ^32^, ^33^, ^34^, ^35^ Patient-level barriers such as unreliable transportation, housing instability, and financial hardship directly limit attendance at follow-up appointments and treatment plan adherence, resulting in worse outcomes and increased complication risk. These challenges are compounded by systemic barriers, as patients with public insurance or greater socioeconomic deprivation often face longer wait times and fewer practices that accept their insurance.^13^, ^36^, ^37^ Specifically for scaphoid fractures, Garala et al^38^ found that patients with the lowest socioeconomic status had nearly double the incidence of scaphoid fractures compared to their higher socioeconomic status peers. When considered alongside our findings and the consequences of delayed diagnosis and treatment, individuals from deprived communities are at significantly higher risk of scaphoid nonunions.^39^ Given this concern, pathways that connect at risk patients with prompt and appropriate management should be developed.

Typical algorithms for suspected scaphoid fractures emphasize early MRI if no fracture is seen on x-ray or CT scan, or if there is a nondisplaced fracture on x-ray, to minimize time to effective treatment.^6^ Murthy et al^40^ examined the role of advanced imaging in scaphoid fractures, finding that early MRI was both clinically and cost effective, resulting in improved patient outcomes and lower costs. The cost-savings of early interventions are especially important for safety-net hospitals caring for uninsured or underinsured patients, as recent Medicaid cuts and under-reimbursement contribute to financial instability.^41^ Beyond advanced imaging, expedited referrals or “fast-track pathways” from the ED to the OR for hip surgeries within the first 24 hours of the injury have been shown to reduce mortality and complications. Protocols like this for scaphoid fractures can mitigate disparities as these patients are cared for immediately.^42^ Although the present study focused on scaphoid fractures, similar access barriers may affect time to evaluation for other nonlife-threatening orthopedic injuries where delays meaningfully worsen outcomes.

This study has several limitations. First, as a retrospective chart review, our study relies on the accuracy and completeness of medical records. Second, although these indices are validated measures of social determinants of health, they may not capture all individual-level socioeconomic factors, such as transportation, health literacy, and access to medical care. Third, our study only included 107 patients, which may limit the statistical power and reduce the precision of effect estimates. Fourth, some patients may have undergone initial assessment elsewhere, which could bias findings toward longer delays. Lastly, as this analysis was conducted at a single institution, findings may not be as generalizable to other institutions or geographic regions. Institutional practice patterns regarding access, particularly for Medicaid-insured patients, may differ by region and could attenuate the observed association between insurance status and delayed presentation in our cohort.

This study created a novel prediction model to determine nonunion status based solely on social determinants of health. These findings support the use of socioeconomic information to guide targeted, system-level interventions (ie, streamlined referral pathways and outreach programs) aimed at reducing delays and mitigating disparities. Future research should be aimed at validating this model with multicenter studies, as well as for other orthopedic injuries.

## Ethics declaration

The authors have NOT obtained informed consent from participants or their legal representatives. Our study is a retrospective chart review. All patient health information were deidentified and data was protected once collected. IRB approval to perform this study without documented consent was acquired through our IRB.

This study was performed in compliance with relevant laws, regulatory frameworks and guidelines where the research took place. The UConn IRB granted an exemption. The authors provided the following explanation: We acquired IRB approval (25X-409-1) for an exempt study for secondary research that consent wasn't required for. All PHI was protected and deidentified.

## Conflicts of Interest

No benefits in any form have been received or will be received related directly to this article.
